# Exploring the Bioactive Content of Liquid Waste and Byproducts Produced by Two-Phase Olive Mills in Laconia (Greece): Is There a Prospect for Added-Value Applications?

**DOI:** 10.3390/foods12244421

**Published:** 2023-12-09

**Authors:** Ioanna Pyrka, Christina Koutra, Vasileios Siderakis, Panagiotis Stathopoulos, Alexios-Leandros Skaltsounis, Nikolaos Nenadis

**Affiliations:** 1 Laboratory of Food Chemistry and Technology, School of Chemistry, Aristotle University of Thessaloniki, 54124 Thessaloniki, Greece; ioannapyrka@chem.auth.gr; 2 Department of Pharmacognosy and Natural Products Chemistry, Faculty of Pharmacy, University of Athens, 15771 Athens, Greece; ckoutral@pharm.uoa.gr (C.K.); billsid@pharm.uoa.gr (V.S.); skaltsounis@pharm.uoa.gr (A.-L.S.)

**Keywords:** olive-mill leaves, olive pomace, two-phase decanter, drying, oleuropein, hydroxytyrosol, oleanolic acid, maslinic acid

## Abstract

The use of a two-phase decanter (TwPD) for olive-oil extraction produces wastes and byproducts (a small volume of water from oil washing, olive leaves from the defoliator, and a high moisture pomace which can be destoned) that contain valuable bioactive compounds, such as phenolics and/or triterpenic acids. So far, there is no (water) or limited information (leaves and the destoned pomace fraction) on their content of bioactives, especially triterpenic acids. To contribute to the characterization of such streams from cultivars of international interest, in the present study, samples obtained from five mills from the region of Laconia (from one or two harvests) in Greece, where Koroneiki cv dominates, were screened for phenols and/or triterpenic acids. The leaves and pomace were dried at two temperatures (70 °C and/or 140 °C), and the pomace was also destoned before analysis. The liquid wastes contained low amounts of total (TPC) phenols (<140 mg gallic acid/L), hydroxytyrosol (<44 mg/L), and tyrosol (<33 mg/L). The olive leaves varied widely in TPC (12.8–57.4 mg gallic acid/g dry leaf) and oleuropein (0.4–56.8 mg/g dry leaf) but contained an appreciable amount of triterpenic acids, mainly oleanolic acid (~12.5–31 mg/g dry leaf, respectively). A higher drying temperature (140 vs. 70 °C) affected rather positively the TPC/oleuropein content, whereas triterpenic acids were unaffected. The destoned pomace TPC was 15.5–22.0 mg gallic acid/g dw, hydroxytyrosol 3.9–5.6 mg/g dw, and maslinic 5.5–19.3 mg/g dw. Drying at 140 °C preserved better its bioactive phenols, whereas triterpenic acids were not influenced. The present findings indicate that TwPD streams may have a prospect as a source of bioactives for added-value applications. Material handling, including drying conditions, may be critical but only for phenols.

## 1. Introduction

Olive mills are an important source of wastes and byproducts, which are obtained in large amounts within their seasonal operation, causing, thus, a major environmental burden. As a matter of fact, around 9.6 million tonnes/year is the estimated biomass deriving from olive mills and around 6 × 10^6^ m^3^ is the corresponding amount of mill wastewater [1,2]. All these materials may contain valuable compounds, with phenols such as hydroxytyrosol (HTY), tyrosol (TY), and/or oleuropein, as well as triterpenic acids, such as maslinic and oleanolic acids, being identified among the major ones to which valuable biological actions have been attributed. Specifically, the beneficial activity of HTY in human health has been approved by the European Food Safety Authority (EFSA), which issued an opinion in favour of a specific health claim, described in EU Regulation 432/2012 [3]. Moreover, recently, the European Union, in a decision (Regulation 2017/2373 of 14 December 2017), approved the use of HTY on the market as a novel food ingredient, in accordance with Regulation of the European Parliament (EC) No. 258/97 [4]. Oleuropein presents an array of biological activities and accounts mostly for the efficiency of olive leaf extracts towards noncommunicable diseases, namely, cardiovascular diseases, cancers, respiratory diseases, and diabetes [5]. Maslinic and oleanolic acids also have a beneficial effect on human health, as they have been shown to exhibit strong antioxidant, antimicrobial, anti-inflammatory, antidiabetic, and anticancer effects [6,7]. Thus, due to the presence of these compounds, and within the framework of the circular economy, there is an increasing trend in the food, feed and pharmaceutical sectors towards the exploitation of olive byproducts and wastes for high-added-value applications [1,2,8,9,10,11,12,13].

Although the approach of using a two-phase decanter (TwPD) may almost diminish the wet effluents, making its adoption rather imperative in the near future due to the increasing environmental legislation constraints [14], some liquid wastes (~0.2 m^3^) are still produced during oil washing [15]. Furthermore, except for the olive leaves deriving from the defoliator and usually stockpiled in the ground, a high-moisture biomass (olive pomace) is obtained which is cumbersome to be disposed of or exploited by the refineries for oil extraction with solvents [16], even after stone removal. Considering the fact that the content/composition of the respective materials is affected by various biotic and abiotic factors, including the postoperational handling, it is evident that screening their content for valuable constituents at different sampling periods may assist decision making and strategic plan development. A more conclusive view can be obtained when the industry or the cooperatives may be involved in the collection and postoperational handling, as it will reveal if there is any prospect for their use and possible bottlenecks which otherwise will remain veiled if expert scientists carry out this part. The need for screening is further supported by the fact that olive-mill leaves have not been widely characterized regarding their bioactive content, contrary to fresh ones collected from the trees [17]. Similar is the case for the two-phase olive-pomace fraction obtained after removing the seed fragments—a relatively new industrial byproduct—which is of potential interest as a source of triterpenic acids [17] and recently as a functional food ingredient [18]. The same applies to the liquid wastes which are neglected as only the wastewater produced in large volumes from the three-phase olive decanters has been extensively studied [17].

Taking into account all these, and the fact that in Greece there has been a transient era since 2011 for the adoption of TwPD, due to legislation restrictions [19] on the treatment of liquid waste, thus, the operation of three-phase decanters (ThPD) is difficult. The present study aims to screen the content of liquid wastes (water from olive-oil washing) and solid/semisolid byproducts (olive leaves, destoned olive-pomace fraction) obtained by such kind of technology, the use of which is steadily increasing in olive mills. Emphasis is given to bioactive phenolic compounds and triterpenic acids from the streams deriving from the processing of the Koroneiki cultivar (or some blends), which is the major one used for olive oil production in Greece but also cultivated in other European and non-European countries [20]. The present findings are expected to be valuable and support the perspective of using these materials as a source of bioactive compounds for added-value applications worldwide.

## 2. Materials and Methods

### 2.1. Materials

The materials examined were liquid wastes from olive-oil washing, olive leaves, and olive pomace, all deriving from two-phase olive mills. The mills (A–Ε) selected were involved in virgin olive-oil production in the region of Laconia (Figure S1). Laconia is one of the major olive-oil-producing regions of Greece in the Peloponnese peninsula, where a wider adaptation of this technology (65%), compared to others such as Crete (35%), is observed at present [21]. The oils produced in 4 out of 5 mills are derived mainly from Koroneiki (80–95%), whereas, in one case, the mill produces oil based on 75% Athenolia and 25% Koroneiki cultivars. The mills, in collaboration with the local union, collected and provided the materials. Specifically, liquid-waste samples were collected in 4 out of 5 of the collaborating mills periodically from December 2021 to February 2022 in bottles of 1 L, with minimum headspace and refrigerated prior to being sent for analysis. Olive leaves were collected from the defoliator during the same operational period, transferred to the union premises and oven-dried at 70 and/or 140 °C. A set of leaves dried at 70 °C was collected in the year 2022–2023 from 3 out of 5 mills (A, C, D). The oven was a commercial one bearing 4 shelves with the dimensions of each shelf being 40 × 60 × 7 cm. In parallel, quantities of olive pomace obtained from different drupe loads, namely mill A: Koroneiki–Athenolia, 25/75%, mill B: Koroneiki–Athenolia, 85/15%, mill C: Koroneiki–Koutsourelia–Athenolia, 85/10/5%, mill D and E: Koroneiki–others, 95/5% were collected and dried at the same two temperatures in the corresponding oven. The dried material was kept in sealed bags (leaves) or plastic sealed bottles (pomace) suitable for human use at room temperature prior to their dispatch to the laboratories for analysis (Figure S2).

### 2.2. Methods

#### 2.2.1. Liquid-Waste Extraction

Liquid waste has passed through a range of sieves with different pore sizes (1 mm, 500 μm, 100 μm, and 45 μm) to remove most of the suspended particles prior to extraction. Then, the liquid was extracted following the protocol proposed by Kalogerakis et al. [22] after some modifications as follows; 200 mL of the sample were transferred to an 800 mL beaker. Subsequently, 400 mL of ethyl acetate were added and then stirred (300 rpm) for 30 min. The glass was covered with watch glass during the extraction to minimise solvent evaporation. The mixture was then transferred to a 1 L separating funnel and left to rest until the two layers were separated. The lower one (aqueous) was extracted once more with 200 mL ethyl acetate. The organic layers derived from the two extractions were combined in a conical flask and anhydrous sodium sulfate was added to remove moisture. The solvent was evaporated under vacuum (40 °C), and the residue was dissolved in 20 mL methanol. The solution was pipetted into a preweighted spherical flask. The solvent was removed to dryness, so as to calculate the yield of extraction. redissolved in 5 mL methanol. and filtered through a PVDF filter with a pore size of 0.22 μm prior to the analyses (Figure S3). A liquid-waste sample from a ThPD included for comparison was treated similarly but redissolved to 20 mL methanol prior to filtration and analyses. Measurements were carried out in triplicate and averaged.

#### 2.2.2. Residual Moisture-Content Determination of Dried Leaf and Pomace Samples

The dried leaves and pomace were examined with regard to the residual moisture content using a Kern and Sohn GmbH (Albstadt, Germany) thermobalance. For such a purpose, 2 g of the sample were placed in an aluminium tray after the balance was zeroed, and the weight was recorded to the nearest 3 decimal places. The sample was heated at a temperature of 105 °C with the aid of a halogen lamp till constant weight. The automatic determination was done in duplicate.

#### 2.2.3. Colour Measurement of Pulverized Dried Leaves and Pomace Samples

The colour of the powdered samples was assessed in terms of the CIE rectangular coordinates L^⁎^ (pure darkness 0/pure lightness 100), a^⁎^ (green −/red +), and b^⁎^ (blue −/yellow +) using a MiniScan XE Plus D/8S Color Analyzer Colorimeter Spectrophotometer (Hunterlab, Reston, VA, USA). The measurement was carried out 5 times and values were averaged.

#### 2.2.4. Extraction of Leaves

One g of dried (70 or 140 °C) and pulverized olive leaves was mixed with 50 mL of methanol, and, then, the mixture was stirred for 30 min at 60 °C according to the European Pharmacopeia protocol [23]. Then, it was centrifuged (ST16R Refrigerated Centrifuge, Thermo Scientific, Waltham, MA, USA) at 10,000× *g* for 10 min. The extraction was carried out in triplicate and the supernatants were combined into a representative one and stored at −20 °C until the analyses.

#### 2.2.5. Dry Fractionation of Olive Pomace

The dried material after milling with a domestic grinder was sieved to obtain a destoned pomace fraction (particle size < 500 µm) and a stone-rich fraction (particle size > 500 µm).

#### 2.2.6. Extraction of the Destoned Pomace and Stone Fractions

One g of dried material was mixed with 20 mL of methanol, and, then, the mixture was transferred to an ultrasonic bath where it remained for 30 min. Then, it was centrifuged (ST16R Refrigerated Centrifuge, Thermo Scientific) at 10,000× *g* for 10 min. The extraction was carried out in triplicate, and the supernatants were combined into a representative one and stored at −20 °C for 24 h to crystalise extracted lipids. Then, the liquid extract was transferred immediately to another tube and kept at −20 °C till the analyses.

#### 2.2.7. Total Phenol Content (TPC) Determination

TPC was measured using the Folin–Ciocalteu colorimetric method [24]: Thus, 5 mL of deionised water were added to a 10 mL volumetric flask and mixed with an appropriate sample amount (0.1–0.5 mL) and 0,5 mL of Folin–Ciocalteu reagent. Exactly 3 min later, 1.5 mL of Na_2_CO_3_ solution (20%) were added, and the mixture was diluted up to 10 mL with deionised water. After one hour in the dark, the absorption was measured at 750 nm against a reference solution. Gallic acid was used as a reference compound. The measurement for each sample was done in triplicate, and the results were expressed as mg GA/L or mg GA/kg extract (liquid waste) and mg GA/kg of dry material (solid byproducts). Each value is the mean of three measurements.

#### 2.2.8. Total Flavonoid Content (TFC) Determination [25]

TFC was measured using an aluminium complexation assay. Thus, 100 μL of AlCl_3_ solution (2% AlCl_3_ in a methanol–acetic acid mixture, 95/5, *v*/*v*) and 1,4 mL of a 5% methanol acetate solution have been successively added to 1 mL of methanolic extract. The absorption of the complex formed at 415 nm was measured against a blank after a time of 30 min. Quercetin was used as a reference compound. The measurement for each sample was done in triplicate and the results (mg QUE/g dry material) were expressed as the average of the three measurements.

#### 2.2.9. Liquid Chromatographic Determination of Hydroxytyrosol (HTY), Τyrosol (TY), and Oleoeuropein (OLE)

The analysis was performed on a Thermo Finnigan HPLC system (Markham, ON, Canada) equipped with a SpectraSystem 1000 degasser, a SpectraSystem P4000 pump, a SpectraSystem AS3000 autosampler, and a UV SpectraSystem UV8000 PDA P detector. Data acquisition was monitored by the ChromQuestTM4.2 software (ThermoScientific^TM^). The quantification was achieved on a reversed-phase Spherisorb Discovery HS C18 column (250 × 4.6 mm, 5 μm, Supelco, Bellefonte, PA, USA) using a mobile phase consisting of 0.2% aqueous orthophosphoric acid (A), methanol (B), and acetonitrile (C) with a flow of 1 mL/min according to the COI proposed method [26]. The gradient elution system was as follows: 0–40 min, 2–25% B, and 2–25% C; 40–45 min, 25–30% B, and 25–30% C; 45–60 min, 30–50% B, and 30–50% C; 60–70 min, 50% B, and 50% C; 70–72 min, 50-2% B, and 50-2% C; 72–82 min 2% B, and 2% C. The injection volume was 20 µL; detection and quantification were carried out at 280 via constructing external calibration curves (y = 2.0784x − 6.2501, R² = 0.9999, (OLE), y = 5.1411x − 5.6472, R² = 0.9995 (HTY), y = 4.8029x − 9.3773, and R² = 0.999 (TY)) using standards of appropriate purity (OLE 98% from Sigma-Aldrich, St. Louis, MO., HTY 98% from Extrasynthese, Genay, France, TY 99.5% from Fluka, Busch, Switzerland). The results were expressed as mg/L (liquid waste) and mg/kg of dry material (solid byproducts). Each value is the mean of triplicate determinations.

#### 2.2.10. Liquid Chromatographic Determination of Maslinic (MA) and Oleanolic (OA) Acids

All analyses were carried out on an Agilent 1260 Infinity II HPLC system, equipped with a quaternary pump and an autosampler, coupled to PDA and ELS detectors in a series. OpenLab CDS software (v.2.4, Agilent, Santa Clara, CA, USA) was used for data acquisition and processing. Separation was achieved on a Waters Spherisorb^®^ ODS2 C18 (5 μm particle size, L × I.D. 25 cm × 4.6 mm) at room temperature. The f was set to 0.8 mL/min. The elution was isocratic using as a mobile phase 0,5% formic acid in water (*v*/*v*) (Eluent A) and acetonitrile (Eluent B) at a ratio of 15:85 *v*/*v*. The total analysis time was 30 min. The injection volume was set at 20 µL. The N_2_ flow was 1.1 L/min, whereas the nebulizer and evaporation temperatures were 75 and 80 °C, respectively. For quantification of MA (98% Sigma Aldrich, St. Louis, MO, USA) and OA (97% Acros Organics BV, Geel, Belgium) external calibration curves were constructed using working solutions in the range of 50–200 ng/µL [ELSD: y = 44.409x − 1436 R^2^ = 0.9986 (MA) and y = 32.196x − 1145.2 R^2^ = 0.9938 (OA)]. The method was applied after inhouse validation to determine the content of the triterpenic acids in dry olive leaves, dry destoned pomace, and the stone-rich fraction. The measurement for each sample was done in triplicate, and the results (mg MA or OA/g of dry material) were expressed as the average of the three measurements.

### 2.3. Statistical Analysis

Statistically significant differences among the mean values from mills obtained during the same harvest year were performed by one-way analysis of variance using the multiple Duncan test at *p* < 0.05. A pairwise comparison of the mean values of samples dried at different temperatures or collected in a different harvest year was carried out via a two-tailed *t*-test at *p* < 0.05. The analyses were carried out with SPSS 14.0 software (SPSS Inc., Chicago, IL, USA).

## 3. Results and Discussion

### 3.1. Liquid-Waste Examination

First, the liquid wastes were examined. They were of ochre or brown colouration depending on the solid content. The one sample obtained from a ThPD was brown and the darkest of all. The yield and total and individual phenol content are provided in Table 1.

Although the statistical analysis indicated that the liquid wastes from mills B and D were rather richer, and the lowest content was observed in those obtained from mill E, it was generally evident that the content of phenols, despite the preconcentration with the aid of extraction, was low. This was clearly obvious via comparison with the corresponding value of the liquid waste obtained from the ThPD. The levels were 12- to ~54-fold lower in the liquids obtained from the four mills. Smaller were the differences when considering the levels determined on an extract basis as evidenced by the corresponding TPC levels. In that case, the T extract was only from 1.4 to 2.7 richer, adding to the fact that the wastes from the four mills were very diluted. Similar observations were made regarding the levels of HTY and TY. Taking into account this evidence, one could say that, with the prerequisite that these liquids are also low in toxic metals, they could be used for the watering of the fields. Nevertheless, taking into account the past study of Papadopoulou et al. [27], where chickens supplied with drinking water containing olive-mill wastewater phenols at a level of 20 and 50 mg gallic acid equivalents/L (corresponding to ~0.1 and ~0.25 mg/L of HTY and TY at each level) had a positive impact on the oxidative stress of the animals, a reconsideration of the exploitation of the respective waste for added-value applications may be worthwhile, with animal breeding being an example. Shimao et al. [28] also described the positive effects of low levels of pure oleuropein (0.1, 0.5 and 2.5 mg/kg) administration on broilers, as well as the data presented in the review of Fotina et al. [29] indicating that supplementation of phenols via drinking water may be more efficient to alleviate stress, as the birds, when being in such condition, reduce significantly the consumption of the feed.

### 3.2. Olive-Leaf Examination

The leaves received were first examined for residual moisture to evaluate the effectiveness of drying on the union premises. In a couple of samples from the first sampling date, a moisture content of 21 and 29%, respectively, was found, whereas, in three others, a content of 10.1 and 11.2% was measured. Eventually, the leaves considered for further examination were those of the second and the final (sixth) sampling, dried at two temperatures, a low (70 °C) and a high (140 °C). The high temperature was selected in view of some findings suggesting that, if applied for a short time, may result in a higher phenol content [24,30,31]. The 140 °C was a high value, but even higher (160 °C) has been applied in the literature [32] and under such conditions. As evident upon the drying of wild olive leaves from Algeria, the oleuropein content of the resulting material did not differ from that of the freeze-dried one. However, the high temperature had an impact on the colour, as the a* value was positive and a loss of ~60% of total chlorophylls was evidenced. The residual moisture of all samples received is given in Table S1. When the aforementioned five samples with moisture content >10% were not taken into account, the measured values were below 8% and even as low as 3.5%, suggesting some thermal abuse. The latter supports the fact that no statistical differences were observed for the effect of drying temperature on the residual moisture of the leaves harvested in the first year (t = 1.6903, df = 4, *p* = 0.1662).

The colour determination (Table 2) indicated that the leaves dried at both temperatures were of poor quality, considering the fact that the a* values were positive in all cases and not negative as they should have been if they were freshly collected and dried ones, e.g., [24], suggesting the significant loss of chlorophyll pigments. Any statistical differences between dried samples from different mills were rather small. An overall visualization using appropriate software [33] indicated a temperature effect, as the leaves dried at the lower temperature were ochre like, whereas those exposed to a high temperature were brown. This was further supported by a mean comparison where a) a high statistical difference was observed between the two treatments regarding the L (t = 37, df = 4, *p* = 0.0001) and b (t = 17.9, df = 4, *p* = 0.0001) values, respectively, but not the *a* (t = 2.54, df = 4, *p* = 0.068) value and b) no difference was observed upon a comparison of samples dried at 70 °C for the two harvest years for any of the colour parameters (t = 3.12–4.12, df = 2, *p* = 0.052–0.089). Heating, especially at high temperatures, may induce browning reactions, and facilitate caramelization and the formation of yellow–brown pheo and pyropheophytins [32]. Cagliari et al. [34], using low temperatures, namely 50 and 70 °C, also reported the formation of yellowish colour in leaves after drying, but this could be due to the long drying times employed (60–75 min). Opposite findings have also been reported [24,30,31] at high temperatures, suggesting the insufficient inactivation of oxidative enzymes when low temperatures (e.g., 60 °C) are applied. It should not be forgotten that losses and browning may occur when leaves are left on the ground to decay under uncontrolled environmental conditions (the usual case in olive mills). Similar also was the quality of the leaves collected in the second year despite the findings of the first year and the communication with the industry.

The 20 samples of dried olive leaves of the first year and those obtained from three mills in the second year were then extracted and analysed with regards to their total phenol and flavonoid content, whereas more specific information was sought for oleuropein and the two main bioactive triterpenic acids, maslinic and oleanolic. The results are provided in Table 3.

Although the quality of the leaves was rather poor, at least on the basis of the colour characteristics, the measurements showed the presence of phenolic compounds employing the F-C test over a wide range (14–57.4 mg GA/g dry leaf) in the leaves of the first year, whereas those of the second year were in the range of 12.8 to 22.2 mg GAE/g dry leaf. Some statistical differences were observed only for phenols in the leaves dried at 70 °C. In those leaves, the average TPC value (~24.1 and 18.9 mg GA/g dry leaf for the first and second years, respectively) was comparable to the corresponding one (12.4 to 27.5 mg GA/g dry leaf using different extraction solvents) reported for leaves of the Picual variety harvested from a mill and extracted after drying at room temperature up to ~7% moisture in the study of Gullon et al. [35]. It was similar also to that found in a recent work of del Mar Contreras et al. [36] (22 mg GA/g of dry leaf), which was obtained after optimization of the extraction process. The high value of phenols (~52 mg GA/g of dry leaf) has been reported only by Marquez et al. [37]. Such value could be ascribed to the technique adopted (homogenizer-assisted extraction at 18,000 rpm) which, after optimization, provided better results than conventional and ultrasound-assisted extraction. Furthermore, the fact that, as stated, the leaves were collected directly from the blowing machine, treated immediately, and dried at >100 °C (washed and dried at 105 °C till the residual moisture was <5%), the corresponding average value for the leaves dried at 140 °C was ~ 38.8 mg GA/g of dry leaf. Although it was 1.61 times higher than that at 70 °C, the content increased by heating significantly only in 6 out of the 10 samples, and only in 3 samples the values were in the range of 50.6 to 57.4 mg GA/g dry leaf, close to the value of Marquez et al. [37]. These differences are probably due to the fact that the collection of olive leaves from the olive mill did not occur under the same conditions. Specifically, the length of time the leaves remain in olive mills until their collection and their storage temperature are two factors that affect the activity of the endogenous enzymes (β-glucosidase, esterase, and polyphenol oxidase) in olive leaves and regulate their phenolic content. In general, when the fresh leaves are dried at high temperatures, the enzymes are deactivated, and, thus, the enzymatic reactions of oxidation and degradation of polyphenols are limited; their composition in phenolic components is increased, therefore. However, if the olive leaves are not collected immediately from the mill and are left for a long time on the ground, the activity of the olive-leaf enzymes increases, and this negatively affects the composition of the phenols in the leaves. In this case, the drying conditions of the leaves at different temperatures do not greatly affect their phenolic content since their enzymes’ activity is very low. Despite all these observations, the statistical treatment of the means of TPC values indicated significant differences (t = 5.1722, df = 4, *p* = 0.0066), suggesting a positive impact at 140 °C. Apart from the above, these values are not necessarily low, as evidenced by a comparison with the literature data for freshly harvested and dried olive leaves e.g., [24,38,39]. Examination of the TFC, which was determined using a selective complexation assay instead of a redox reaction applied for total phenols (F-C), showed that the difference between the two olive-leaf thermal treatments was negligible (mean value 3.94 and 3.41 mg quercetin/g dry leaf, respectively). This was also evidenced by applying a *t*-test (t = 1.9021, df = 4, *p* = 0.1299). The levels for total flavonoids were not comparable to those reported in the literature due to the application of different protocols and reference compounds. However, in the work of Papoti et al. [39], where the same protocol and reference compound were applied to extracts from the leaves of some uncommon Greek varieties that are not very rich in total phenols (F-C), the values for flavonoids were much higher. Particularly, in eight out of the nine samples, levels from 11 to 22 mg quercetin/g dry leaf were reported and only in one sample was the value 4.9. In our samples, the maximum value was around 5.5 mg quercetin/g dry leaf. The oleuropein content was more informative regarding the quality of olive leaves. A large variation was found, with the values being in the range from 0.4 to 56.8 mg/g of dry leaf. In this respect, it is worth noting that del Mar Contreras et al. [36], following the optimization of extraction from the olive mill leaves, reported an OLE level of ~4.2 mg/g of dry leaves, whereas Marquez et al. [37], probably for reasons discussed above, found a value of 43 mg/g of dry leaf. Most of these values are well below the range (10–140 mg/g) reported for the olive leaves in the literature [40]. Although the average value for those dried at 140 °C was ~3 times higher than that of leaves dried at 70 °C, the variation was large, and eventually, for 14 out of 20 samples, the oleuropein level was less than 9.2 mg/g. Statistical analysis of the mean values showed a temperature effect, (t = 3.0095, df = 4, *p* = 0.1299) which may justify the findings for TPC, granted that no influence was observed for flavonoids. The present findings, considering the acidic smell in most of the samples, suggest rather some delay in treating the leaves, or even left in the ground (considering the dust found), facilitating, thus, the activity of oxidative enzymes, which can be active even upon decay of the leaves [18]. On the other hand, the levels of the triterpenic acids fluctuated less and were considerable, despite the bad condition of the leaves. Specifically in all the samples examined, the levels of MA were between 3 and 5 mg/g dry leaf, and those of the more abundant OA were in the range of 12 to 31 mg/g dry leaf. As a matter of fact, in many of the samples, the content of triterpenic acids, especially the one of OA, was more than that of OLE. The present findings verify the observation of Romero et al. [17] that olive leaves, even collected from the ground, can be a good source of triterpenic acids, although the phenol content could be negligible. It should also be stressed that triterpenic acids may withstand heating, considering that the melting point is high. As shown by Fulias et al. [41], the decomposition of OA upon thermal analysis was achieved at 286 °C. Consequently, these compounds may survive upon the thermal abuse of the leaves under the adopted drying conditions. Indeed, no temperature effect was observed for both maslinic (t = 0.3362, df = 4, *p* = 0.7336) and oleanolic (t = 0.7616, df = 4, *p* = 0.4887) acids by applying statistics. On the other hand, oleuropein could possibly decay upon extensive heating, as its m.p. is much lower (~90 °C) [42]. The levels reported in the present study were of the same size as those found by Romero et al. [17,43] for Picual leaves and higher than those measured in leaves from Arbequina. Similarly, the present findings were also higher than those reported by Xie et al. [44] for dried leaves from Frantoio, Leccino, and Moraiolo. No influence of the harvest year was evident at any of the parameters measured (t = 0.6724–2.2491, df = 2, *p* = 0.1535–0.5706).

### 3.3. Destoned Olive-Pomace Fraction Examination

The olive-pomace samples were dried at the same temperatures applied to the olive leaves, with the high one also being used in the literature for the same material (TwPD olive pomace) [45]. After the drying process, the samples were characterised by low luminosity and, considering the values a and b, appeared dark brown (Table 4). Some rather small statistical differences were observed among the samples dried at 140 °C. Those dried at 70 °C, due to technical issues, were too few in number to conclude whether there was or not an effect on colouration, as it was evident for the olive leaves. The additional samples obtained in the second year and dried at 70 °C were rather closer in colour to those dried at 140 °C from the first year. Thus, no clear effect on pomace colour vs. drying temperature could be deduced. This was verified by the lack of statistical significance when comparing the means (t = 1.6705–3.6422, df = 2, *p* = 0.0678–0.2368) of the samples harvested in the first year. No comparison was made with the second-year harvest, as only one sample (C) was available from the same mill.

The residual moisture (Table S1) was very low, lower than 5%, suggesting extensive exposure to heat under both conditions employed. The yield of extraction from the destoned pomace fraction was similar for the material dried at both temperatures (Table 5), and it was lower than that of the olive leaves.

There were almost no statistical differences among samples from the olive mills. The total phenol content of the samples from the first year was on average 20.4 mg/kg of dry destoned pomace, a value close to the average of the leaves dried at 70 °C (just ~1.18 times lower) but ~1.9 times lower than that of the leaves dried at 140 °C. The average value for the first year of the dried samples at 140 °C was similar to those of two corresponding samples of crude pomace from different mills (~20 and 22 mg/g of dry olive pomace) in the study of Ribeiro et al. [19]. Moreover, it was greater than the dry destoned pomace fractions (~9 and 15 g/kg dry olive kernel) produced by the authors with sieving, as, prior to drying, part of the water containing a significant amount of phenols had been removed via centrifugation. The content was also similar to that reported in the study of Cravotto et al. [46] (19.7 mg/g dry olive pomace). It is worth noting that the content of the same material tested after drying at 70 °C was about 50% lower (t = 7.3523, df = 2, *p* = 0.0180), although no colour differences between the two types of samples were observed. Similar observations were made for those samples obtained in the second year and dried at 70 °C, despite the observations of the first year. The quantitative differences could be partially attributed to the formation of new phenolic compounds at high temperatures (90–150 °C) due to nonenzymatic interconversions leading to the availability of precursor phenolic compounds [30], which was also observed in other plant materials [47]. Another reason could be the ineffective prevention of the enzymatic activity at low temperatures due to the slow drying process. Examination of some of the samples showed that the material contained a too-low amount of flavonoids (0.4–0.5 mg que/g dry destoned pomace), which was rather expected when taking into account the literature [17]. Thus, no measurements were carried out in all samples. The observation made in terms of total phenol content about the lower quality of the three samples dried at 70 °C was confirmed via HPLC analysis and the determination of HTY and TY contents (t = 9.4296, *p* = 0.0111, t = 10, *p* = 0.0099). Regarding the former, the levels ranged from 2.3 to 8.5 mg/g (m. value 4.4 ± 1.7 mg/g) and those of the latter from 0.3 to 1.5 mg/g (m. value 0.7 ± 0.3 mg/g). In the study of Ribeiro et al. [18], the two olive-pomace samples, before any fractionation, had a content of 2.0 and 1.7 mg/g in HTY and 0.5 and 0.6 mg/g in TY. As a result of the removal of most of the water via centrifugation, the examination of the two destoned pomace fractions showed levels of 0.8 and 0.3 g/kg, which is almost 10-fold lower than those in the present study, suggesting that water should not be removed if a rich material in biophenols is desired. Terpenoid analysis showed that the destoned pomace fraction contained higher levels of MA than HTY, and OA was higher than TY.

Subsequently, it was attempted to study the stone-rich fraction so as to evaluate how effective the fractionation approach we followed was. As the size was large and irregular, further grinding was carried out, and the content of total phenols, before and after grinding, was measured. As an extraction protocol, the same that was applied to the destoned pomace was used. Total phenol content increased, as evidenced in a random sample from 5.4 ± 0.1 to 7.3 ± 0.1 mg GA/g stone. As a consequence, grinding and sieving followed. The study actually showed that this material was poor in total and individual phenols (Table 6), as the mean value was 6.0 ± 0.8 (TPC), 1.0 ± 0.3 (HTY), and 0.21 ± 0.1 (TY) mg/g, respectively. In a few of these samples analysed, MA was found in the range of 0.4–2.4, and OA was 0.2–1.0 mg/g. Furthermore, a low % extraction yield was found. Thus, the fractionation approach was rather successful despite some statistical differences among the samples of different mills. The stone fraction, which was poor in bioactives, could be a candidate, as shown by Ribeiro et al. [18], for use as a biofuel based on its high calorific value.

Therefore, by targeting the bioactive phenols of the pomace, drying the material at a relatively high temperature followed by dry fractionation can then lead to a relatively rich material, especially in HTY and MA, which is the main phenol and main triterpenic acid identified in the pomace, respectively. To avoid dry fractionation, it seems that the separation of the stone can be achieved by industrial sieving directly in the wet pomace [48], a practice highlighted in Spain by Romero et al. [17] leading to a new byproduct. Such a material is expected to be safe and, in addition to the bioactive phenols and terpenoids, should contain an appreciable amount of dietary fiber [49].

## 4. Conclusions

The present screening study of the waste and byproducts from the region of Laconia deriving from mills with two-phase decanter technology, processing mainly Koroneiki cv, showed that they can be useful for high-added-value applications despite some bottlenecks regarding handling by the local industry. The liquid waste (water from oil washing), which to our knowledge has not been studied in the past, though diluted, contains hydroxytyrosol (<44 mg/L) and tyrosol (<33 mg/L) at levels that could, for example, have a beneficial effect in animal breeding. This may occur via its supplementation as ‘drinking’, with or without dilution, after some filtration to reduce solids, and, definitely if its microbiological and chemical safety will be verified. The mill leaves may be used as a source of bioactive compounds; despite the instability of phenols due to handling issues and drying temperature, which may cause a large variation in their levels (14–57.4 mg GA/g dry leaf), can serve, even in case oleuropein is low (<10 mg/g dry leaf) or even negligible (0.4 mg/g dry leaf), as a source of bioactive triterpenoids, namely of OA (12.6–26.7 mg/g dry leaf). As a matter of fact, the leaves were richer in terpenoids even from the destoned pomace fractions examined, which were rich in MA (5.5–13.2 mg/g dry destoned pomace). In the case of the pomace, the proposed fractionation approach adopted from the literature could lead to a material enriched in bioactives, which is candidate even for food use, whereas the stone-fraction for biofuel production gives possible ideas for additional income. The use of emerging techniques for drying (e.g., infrared and microwave) could facilitate a faster process and thus, permit the handling of larger amounts of byproducts and the use of inexpensive thermobalances to monitor the drying process. The latter could help to avoid sample overheating and could possibly solve some technical problems. Such approaches may lead to the production of materials with higher content in bioactive phenols as triterpenic acids were stable regardless of the condition of the leaves or the drying temperature. All these should be proven in practice with a systematic analysis of the appropriate samples over the course of years in the future.

## Figures and Tables

**Table 1:** Extraction yield, total phenol (TPC), hydroxytyrosol (HTY), and tyrosol (TY) content of water from olive-oil washing step collected from different olive mills with two-phase decanters at different operation periods (harvest period 2021–2022).

Samples	Extraction Yield % (mg/100 mL) *	TPC (mg GA/L) *	TPC (mg GA/kg Extract) *	HTY (mg/L) *	TΥ (mg/L) *
A	36.5 ± 28.0	49.4 ± 46.2 ^a^	118.0 ± 39.7 ^a^	15.3 ± 13.3 ^a^	13.8 ± 6.6 ^a^
B	58.5 ± 34.3	88.7 ± 47.6 ^a,b^	154.2 ± 35.7 ^a,b^	22.8 ± 13.0 ^a,b^	17.5 ± 6.5 ^a^
D	59.4 ± 29.9	76.2 ± 12.0 ^a,b^	142.7 ± 34.2 ^a,b^	28.9 ± 15.2 ^a,b^	26.0 ± 7.4 ^b^
E	20.6 ± 17.2	20.6 ± 23.5 ^a,c^	78.5 ± 36.8 ^a,c^	3.1 ± 2.3 ^a,c^	4.5 ± 5.9 ^c^
ThPD	510.6	1073.3	210	637	162.3

A, B, D, E: olive mills with two-phase decanter; ThPD: olive mill with three-phase decanter; GA: gallic acid; * Values (except for ThPD) are means of 6 samplings within a 4-month period of operation analysed in triplicate (*n* = 6 × 3) ± SD. Values in the same column with different superscripts differ significantly (*p* ≤ 0.05).

**Table 2:** Colour coordinates (L, a, b) of ground olive leaves (2021–2022 and 2022–2023 harvest periods) dried at 70 and/or 140 °C.

Samples	Colour Coordinates of Ground-Dried Leaves					
	L*	a*	b*	L*	a*	b*
2021–2022		70 °C **			140 °C **	
A	60.8 ± 4.5	5.8 ± 0.3 ^a^	31.0 ± 0.6 ^a^	29.7 ± 1.2	3.7 ± 1.0	15.3 ± 0.5
B	58.2 ± 6.1	6.4 ± 1.1 ^a^	33.2 ± 0.0 ^a,b^	29.0 ± 0.4	3.9 ± 1.1	14.7 ± 0.1
C	61.4 ± 0.2	5.7 ± 0.9 ^a^	37.1 ± 1.2 ^b^	31.3 ± 0.3	4.2 ± 1.2	15.8 ± 1.4
D	55.7 ± 2.9	5.3 ± 1.4 ^a^	34.0 ± 3.8 ^a,b^	27.2 ± 2.9	3.6 ± 0.6	13.4 ± 4.5
E	62.7 ± 0.3	2.9 ± 0.1 ^b^	37.2 ± 2.6 ^b^	29.5 ± 0.5	3.6 ± 0.4	15.8 ± 2.8
2022–2023		70 °C ***				
A	48.1 ± 1.2	3.1 ± 0.2 ^a^	27.8 ± 1.1			
C	43.8 ± 3.6	4.8 ± 0.9 ^b^	27.1 ± 0.4			
D	48.4 ± 0.4	3.0 ± 0.2 ^a^	28.5 ± 0.4			

A–E: mills with two-phase decanter; Values are means of 2 samplings within a 4-month period of operation analysed in triplicate (*n* = 2 × 3) ± SD. Values in the same column per treatment with different superscripts differ significantly (*p* ≤ 0.05). ** collected in 2021–2022 harvest period. *** collected in 2022–2023 harvest period.

**Table 3:** Yield of extraction, total phenol (TPC), total flavonoid (TFC), oleuropein (OLE), maslinic (MA), and oleanolic (OA) acids content of olive leaves (2021–2022 and 2022–2023 harvest periods) dried at 70 and/or 140 °C.

Samples	Yield of Extraction (%) *	TPC (mg GA/g Dried Leaf) *	TFC (mg QUE/g Dry Leaf) *	OLE (mg/g Dry Leaf) *	MA (mg/g Dry Leaf) *	OA (mg/g Dry Leaf) *
2021–2022			70 °C **			
A	23.7 ± 9.3	17.2 ± 4.0 ^a^	3.4 ± 0.6 ^a^	3.0 ± 3.3 ^a^	4.4 ± 1.4	25.0 ± 6.1
B	29.9 ± 4.2	21.6 ± 2.3 ^a,b^	3.5 ± 0.2 ^a^	1.3 ± 0.5 ^a^	3.5 ± 0.0	20.4 ± 0.1
C	33.7 ± 4.8	26.0 ± 12.0 ^a,b^	3.9 ± 0.1 ^a^	4.0 ± 2.5 ^a^	4.1 ± 0.0	21.8 ± 0.7
D	26.2 ± 6.6	21.5 ± 5.6 ^a,b^	4.0 ± 0.9 ^a^	3.7 ± 4.4 ^a^	3.8 ± 0.2	21.4 ± 1.6
E	33.1 ± 10.0	34.2 ± 5.8 ^b^	5.1 ± 0.6 ^b^	18.2 ± 12.7 ^b^	4.2 ± 0.7	24.2 ± 2.4
2021–2022			140 °C **			
A	25.8 ± 13.0	41.8 ± 1.7	3.6 ± 1.3	12.4 ± 14.9	2.9 ± 1.0 ^a^	16.4 ± 4.0
B	29.1 ± 12.0	34.3 ± 14.1	3.3 ± 2.2	13.0 ± 14.8	3.9 ± 0.3 ^b^	21.1 ± 0.1
C	30.0 ± 18.7	40.9 ± 23.3	3.4 ± 2.2	29.1 ± 39.2	4.5 ± 0.3 ^b^	23.0 ± 2.2
D	27.9 ± 16.1	36.2 ± 23.5	3.3 ± 2.2	20.6 ± 28.5	4.0 ± 0.4 ^b^	22.1 ± 0.5
E	31.3 ± 11.0	41.1 ± 13.5	3.6 ± 1.7	18.2 ± 18.0	4.1 ± 0.9 ^b^	23.2 ± 3.5
2022–2023			70 °C ***			
A	24.2 ± 2.0	19.7 ± 1.8 ^a^	4.3 ± 0.3 ^a^	4.6 ± 1.7	3.1 ± 0.9	16.5 ± 3.9
C	27.1 ± 7.3	15.4 ± 3.0 ^b^	3.6 ± 0.2 ^b^	3.9 ± 2.6	3.9 ± 0.1	19.4 ± 1.5
D	26.9 ± 0.9	21.5 ± 0.7 ^a^	4.8 ± 0.3 ^a^	7.2 ± 0.6	3.4 ± 0.0	18.8 ± 0.2

A–E: mills with two-phase decanter; GA: gallic acid, QUE: quercetin. * Values are means of 2 samplings within a 4-month period of operation analysed in triplicate (*n* = 2 × 3) ± SD. Values in the same column per treatment with different superscripts differ significantly (*p* ≤ 0.05). ** collected in 2021–2022 harvest period. *** collected in 2022–2023 harvest period.

**Table 4:** Colour coordinates (L, a, b) of olive pomace (2021–2022 and 2022–2023 harvest periods) dried at 70 and/or 140 °C.

Samples/Harvest Period	L*	a*	b*
2021–2022		140 °C	
A	19.5 ± 1.2 ^a^	4.6 ± 0.8 ^a^	6.7 ± 1.4 ^a^
B	19.5 ± 1.0 ^a^	5.2 ± 0.5 ^a^	6.9 ± 1.1 ^a^
C	18.2 ± 1.0 ^a^	4.9 ± 1.1 ^a^	5.7 ± 1.7 ^a^
D	19.9 ± 0.9 ^a^	5.7 ± 0.4 ^a,b^	7.7 ± 1.2 ^a^
E	15.9 ± 0.9 ^b^	2.7 ± 0.8 ^c^	2.6 ± 1.6 ^b^
2021–2022		70 °C	
A	29.5	6.7	13.6
C	21.6	4.7	6.3
D	27.7	7.3	12.3
2022–2023		70 °C	
B	20.6 ± 1.8 ^a^	5.1 ± 1.3 ^a^	7.6 ± 1.5 ^a^
C	16.2 ± 1.4 ^b^	2.4 ± 0.8 ^b^	3.4 ± 1.0 ^b^
E	16.5 ± 2.0 ^b^	2.8 ± 1.0 ^b^	3.4 ± 0.9 ^b^

A–E: olive mills with two-phase decanter; Values are means of 6 samplings within a 4-month period of operation analysed in quintuple (*n* = 6 × 5) ± SD. Values in the same column per treatment with different superscripts differ significantly (*p* ≤ 0.05).

**Table 5:** Yield of extraction, total phenol (TPC), hydroxytyrosol (HTY), tyrosol (TY), oleuropein (OLE), maslinic (MA), and oleanolic (OA) acids content of destoned olive-pomace fraction (2021–2022 and 2022–2023 harvest periods) obtained from pomace dried at 70 and/or 140 °C.

Samples/Harvest Period	Yield of Extraction (%) *	TPC (mg GA/g Dry Destoned Pomace) *	HΤY (mg /g Dry Destoned Pomace) *	ΤY (mg/g Dry Destoned Pomace) *	MA (mg/g Dry Destoned Pomace) *	OA (mg/g Dry Destoned Pomace) *
2021–2022			140 °C			
A	18.1 ± 1.8	17.9 ± 3.0	4.5 ± 1.9	1.0 ± 0.3	11.1 ± 2.0 ^a^	4.5 ± 0.7 ^a^
B	18.5 ± 4.0	23.0 ± 3.1	4.6 ± 2.0	0.6 ± 0.2	8.7 ± 3.6 ^a,b^	1.4 ± 0.6 ^b^
C	20.3 ± 5.1	22.9 ± 2.3	5.6 ± 1.8	0.7 ± 0.2	6.9 ± 1.4 ^b^	3.1 ± 0.5 ^c^
D	16.8 ± 4.3	18.9 ± 1.3	3.6 ± 1.2	0.5 ± 0.2	8.7 ± 3.5 ^a,b^	3.5 ± 1.2 ^a,c^
E	15.5 ± 3.3	18.2 ± 4.1	3.9 ± 1.3	0.7 ± 0.1	10.4 ± 2.8 ^a,b^	4.2 ± 0.9 ^a^
2021–2022			70 °C			
B	15.9	11.8	0.3	0.3	8.2	3.3
C	22.2	11.2	0.4	0.3	8.2	3.6
E	20.0	10.5	0.3	0.4	9.4	3.6
2022–2023			70 °C			
A	19.6 ± 5.8	9.0 ± 2.0 ^a^	0.8 ± 0.3 ^a^	0.3 ± 0.3	13.0 ± 6.3	5.6 ± 2.8
C	18.1 ± 3.9	4.6 ± 0.7 ^b^	0.1 ± 0.0 ^b^	0.2 ± 0.0	13.0 ± 2.2	5.1 ± 0.7
D	10.8 ± 3.3	7.4 ± 1.4 ^a^	0.5 ± 0.2 ^a^	0.2 ± 0.2	9.0 ± 1.3	3.5 ± 0.3

A–E: olive mills with two-phase decanter; * Values are means of 6 samplings within a 4-month period of operation analysed in triplicate (*n* = 6 × 3) ± SD. Values in the same column per treatment with different superscripts differ significantly (*p* ≤ 0.05).

**Table 6:** Yield of extraction, total phenol (TPC), hydroxytyrosol (HTY), and tyrosol (TY) content of olive-pomace stone-rich fraction (2021–2022, 2022–2023 harvest periods) obtained from dried pomace at 70 and/or 140 °C.

Samples/Harvest Period	Yield of Extraction (%) *	TPC (mg GA/g Dry Stone) *	HΤY (mg /g Dry Stone) *	ΤY (mg/g Dry Stone) *
2021–2022		140 °C		
A	2.6 ± 0.7 ^a^	5.3 ± 0.8	1.0 ± 0.5	0.2 ± 0.1
B	3.8 ± 1.1 ^a,b^	6.2 ± 0.6	1.0 ± 0.3	0.2 ± 0.0
C	4.6 ± 0.9 ^b^	6.6 ± 0.7	1.2 ± 0.3	0.2 ± 0.1
D	4.2 ± 0.9 ^b^	6.0 ± 0.6	1.0 ± 0.4	0.2 ± 0.1
E	4.2 ± 0.8 ^b^	6.2 ± 0.7	1.0 ± 0.2	0.2 ± 0.1
2021–2022		70 °C		
B	3.4	3.0	0.1	0.1
C	7	3.9	0.1	0.2
E	6.6	3.2	0.1	0.1
2022–2023		70 °C		
A	2.0 ± 0.3 ^a^	4.3 ± 0.8	<0.1	<0.06
C	6.5 ± 2.0 ^b^	5.2 ± 0.5	<0.2	<0.1
D	6.9 ± 3.0 ^b^	4.8 ± 1.0	<0.4	<0.2

A–E: mills with two-phase decanter; GA: gallic acid; QUE: quercetin. * Values (except for B, C, E, 2021–2022) are means of 6 samplings within a 4-month period of operation analysed in triplicate (*n* = 6 × 3) ± SD. Values in the same column per treatment with different superscripts differ significantly (*p* ≤ 0.05).

## Data Availability

The data presented in this study are available on request from the corresponding author.

## Supplementary Materials

The following supporting information can be downloaded at: https://www.mdpi.com/article/10.3390/foods12244421/s1, Figure S1: Olive mills with two-phase decanter (A–E) and EASL location in Laconia region; Figure S2: Sampling scheme, Figure S3: Extraction scheme of phenols from liquid waste; Table S1: Residual moisture content of leaves and olive pomace (2021–2022 and 2022–2023 harvest periods) dried at 70 and/or 140 °C.
