# Supplementary material for: Exploring the Bioactive Content of Liquid Waste and Byproducts Produced by Two-Phase Olive Mills in Laconia (Greece): Is There a Prospect for Added-Value Applications?
Source: Foods. 2023 Dec 9;12(24):4421. doi: 10.3390/foods12244421 (PMC10742542; doi:10.3390/foods12244421)
Supplement: Supplementary file 1 [file foods-12-04421-s001.zip › foods-2734098-supplementary.pdf]

SUPPLEMENTARY MATERIAL

Exploring the Bioactive Content of Liquid Waste and Byproducts Produced by Two-Phase Olive Mills in Laconia (Greece): Is There a Prospect for Added-Value Applications?

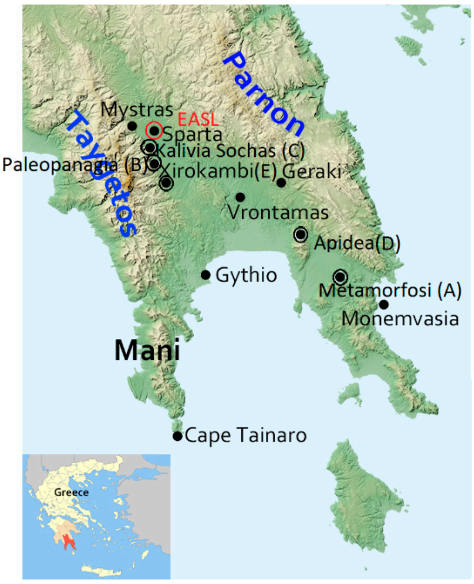

Figure S1. Olive mills with two-phase decanter (A-E) and EASL location in Laconia region.

Table S1. Residual moisture content of leaves and olive pomace (2021-2022, 2022-2023 harvest periods) dried at 70 and/or 140 °C

	Residual moisture content					
**samples**	**Olive Leaves**			**Olive Pomace**		
	70 °C*/**	140 °C*/**	70 °C*/***	140 °C*/**	70 °C*/**	70 °C*/***
A	7.4±7.1	8.0±10.4	8.1±0.1	2.2±1.3	-	8.8±1.8[a]
B	4.5±1.2	4.3±1.4	-	2.1±0.9	3.7	-
C	4.7±1.2	3.5±1.4	8.4±0.2	3.0±2.3	4.8	5.6±0.7[b]
D	6.4±2.9	4.2±2.9	8.1±0.3	1.7±0.7	-	8.1±1.1[a]
E	5.0±3.1	4.0±1.7	-	2.8±1.8	5.2	-

A-E: mills with two-phase decanters,

*Values (A-E) are means of 6 samplings within a 4 month period of operation analyzed in triplicate (*n*= 6x3) ± SD. Values in the same column with different superscripts differ significantly ($p \leq 0.05$).

** collected at 2021-2022 harvest period

***collected at 2022-2023 harvest period

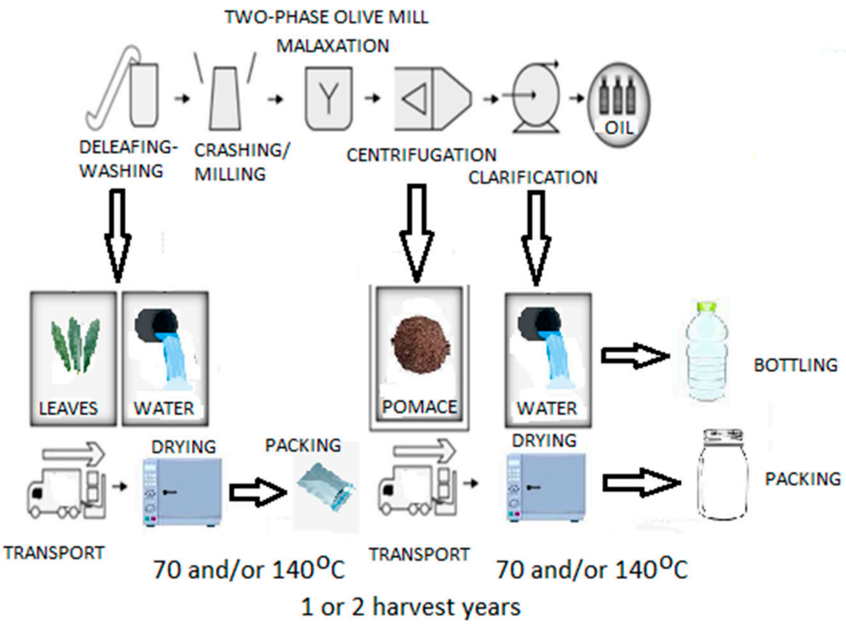

Figure S2. Sampling scheme.

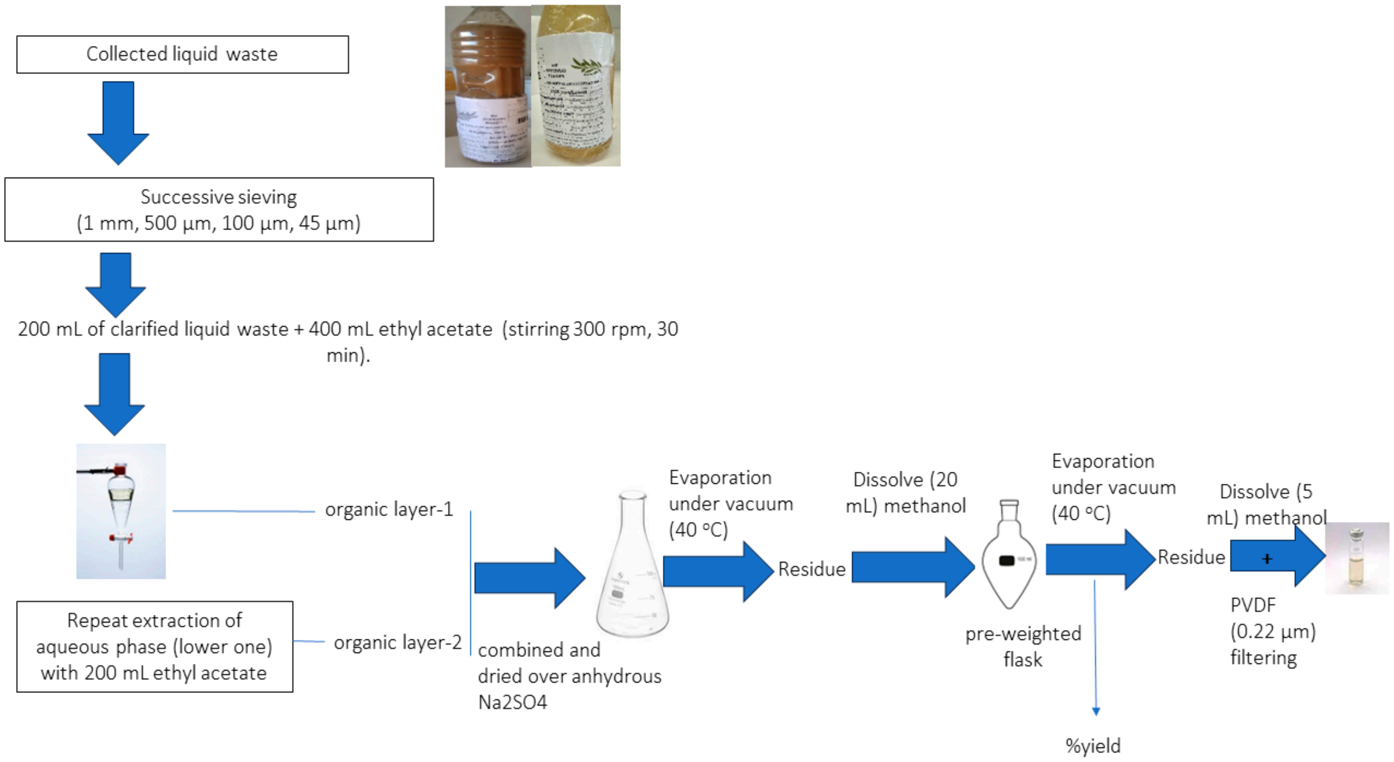

Figure S3. Extraction scheme of phenols from liquid waste.
